# Bifidobacteria isolated from vaginal and gut microbiomes are indistinguishable by comparative genomics

**DOI:** 10.1371/journal.pone.0196290

**Published:** 2018-04-23

**Authors:** Aline C. Freitas, Janet E. Hill

**Affiliations:** Department of Veterinary Microbiology, University of Saskatchewan, Saskatoon, Saskatchewan, Canada; Robert Koch Institute, GERMANY

## Abstract

Bifidobacteria colonize the human gastrointestinal tract, vagina, oral cavity and breast milk. They influence human physiology and nutrition through health-promoting effects, play an important role as primary colonizers of the newborn gut, and contribute to vaginal microbiome homeostasis by producing lactic acid. Nevertheless, the mechanisms by which bifidobacteria are transmitted from mother to infant remains in discussion. Moreover, studies have suggested that *Bifidobacterium* spp. have specializations for gut colonization, but comparisons of strains of the same bifidobacteria species from different body sites are lacking. Here, our objective was to compare the genomes of *Bifidobacterium breve* (n = 17) and *Bifidobacterium longum* (n = 26) to assess whether gut and vaginal isolates of either species were distinguishable based on genome content. Comparison of the general genome features showed that vaginal and gut isolates did not differ in size, GC content, number of genes and CRISPR, either for *B*. *breve* or *B*. *longum*. Average nucleotide identity and whole genome phylogeny analysis revealed that vaginal and gut isolates did not cluster separately. Vaginal and gut isolates also had a similar COG (Cluster of Orthologous Group) category distribution. Differences in the accessory genomes between vaginal and gut strains were observed, but were not sufficient to distinguish isolates based on their origin. The results of this study support the hypothesis that the vaginal and gut microbiomes are colonized by a shared community of *Bifidobacterium*, and further emphasize the potential importance of the maternal vaginal microbiome as a source of infant gut microbiota.

## Introduction

*Bifidobacterium* are Gram-positive, non-motile, anaerobic, non-spore forming rod-shaped bacteria. They belong to the *Bifidobacteriaceae* family and are characterized by high genomic G+C content (55–67 mol%) [1]. Bifidobacteria are common members of the gastrointestinal tract (GIT), representing ~10% of the adult gut microbiota [2, 3]. Bifidobacteria also colonize the human vagina, oral cavity, and breast milk [1, 4]. Beyond the human microbiome, they can be found in sewage, fermented milk products, and the gastrointestinal tracts of animals including insects [5]. Although members of the genus inhabit a wide range of habitats, most *Bifidobacterium* spp. are host-specific [1].

Bifidobacteria have been the subject of numerous studies due their probiotic potential and health promoting characteristics, such as immune modulation [6, 7], production of bacteriocins [8] and inhibition of pathogens [9–11]. The precise mechanisms by which bifidobacteria provide these benefits, however, are not fully understood. Bifidobacteria also play an important role as one of the primary colonizers of the neonatal gut, representing 60–91% of fecal bacteria in breast-fed infants [12, 13]. This early microbial colonization is an essential step in the modulation of the neonatal immune system [14, 15] and may be influenced by mode of delivery (vaginal or C-section) and feeding type (breast milk or formula) [16–18].

Studies of the vaginal microbiota using deep sequencing methods have shown that bifidobacteria are the dominant bacteria in the vaginal microbiomes of some reproductive aged women [19]. Culture-based studies have subsequently confirmed that some vaginal *Bifidobacterium* spp. (Actinobacteria phylum) are able to perform a protective role similar to the beneficial lactobacilli (Firmicutes phylum), which includes the production of lactic acid and hydrogen peroxide [19, 20]. These features prevent the overgrowth of unwanted bacteria and help to maintain the homeostasis of the vaginal microbiome. Common species detected in the vaginal microbiome, based on sequencing [19, 21, 22] and culture methods [19, 23], include *Bifidobacterium breve*, *B*. *longum*, *B*. *bifidum*, and *B*. *dentium*. Based on the recognition that both the gut and vagina harbour bifidobacteria, a large number of studies have been conducted to investigate and demonstrate the influence of maternal microbiota on the neonatal gut microbiome [4, 17, 18, 24–30], but the specific contribution of each microbial community (vaginal, gut and to a smaller extent, milk) in the mother-to-infant bifidobacteria transmission remains in discussion.

Comparison of *Bifidobacterium* spp. genome sequences has revealed a high degree of conservation and synteny across their genomes [31]. Nevertheless, phenotypic differences have been described among bifidobacteria species indicating some degree of species adaptation [32]. One indication of these adaptations is the greater percentage of the bifidobacteria genome involved in carbohydrate metabolism in comparison with the genomes of other members of the gut microbiota. Specifically, genome analysis has demonstrated that *B*. *longum* has the ability to metabolize a variety of complex sugars, which gives an ecological advantage in the GIT and evidently reflects its gut adaptation [33]. It has also been shown that *B*. *longum* subsp. *infantis* has adaptations for milk utilization [34], and that *B*. *dentium* is adapted for the colonization of the human oral cavity [35]. These observations, however, mostly reflect the differences among different bifidobacteria species rather than among strains of the same species from different ecological niches.

The importance of bifidobacteria in adult and neonatal health is evident, although evidence supporting the importance of vertical transmission of maternal microbiota in establishing these populations remains inconclusive. The overlapping occurrence of bacterial species in different body sites is one of the challenges in studying vertical transmission. While several bifidobacteria adaptations for survival in the GIT and oral cavity have been proposed, no study has addressed possible adaptations to the genital tract, in particular, by comparing strains of the same species from different body sites. Here, we compared the genomes of gut and vaginal isolates of *B*. *breve* and *B*. *longum* to identify evidence of strain specialization that could indicate if vaginal and gut strains represent two distinct, adapted subpopulations. Improved knowledge of bifidobacteria ecology is necessary for a better understanding of mother-to-infant bifidobacteria transmission, a potentially important determinant of infant health.

## Material and methods

### Bacterial strains

A total of 16 bifidobacteria (7 *Bifidobacterium breve* and 9 *Bifidobacterium longum*) were sequenced in this study. Genome sequences from an additional 27 *Bifidobacterium* spp. were acquired from GenBank for comparative analysis (Table 1). All 16 genomes sequenced in this study were from strains originally isolated from human vaginal microbiota as part of previous studies [19, 20]. Gut and vaginal isolates were from different individuals. Genomic DNA was isolated from cultures grown in Modified Reinforced Clostridial broth using a modified salting out procedure [36]. The integrity of DNA was verified by electrophoresis on 1% agarose gels. Genomic DNA was quantified using Qubit dsDNA BR assay kit (Invitrogen, Burlington, Ontario) and DNA quality was assessed by the A_260_/A_280_ ratio using a spectrophotometer.

**Table 1:** General features of the bifidobacteria genomes included in this study.

Strain	Ecological origin	Size (Mb)	GC (%)	Total genes	tRNA	CRISPR	N50 / N90	GenBank	Status (n. scaffolds/ contigs)
***B*. *breve* (n = 17)**									
B.b.^#^ 30–1	Vagina	2.54	59.8	2346	67	1	125268 / 38442	*	D (32 / 5)
B.b. 91–1	Vagina	2.24	58.0	2215	50	0	188169 / 56499	*	D (21 / 1)
B.b. 322–1	Vagina	2.24	58.5	2026	47	1	29270 / 9437	*	D (67 / 36)
B.b. W20-13	Vagina	2.30	58.3	2113	50	1	43276 / 19775	*	D (49 / 16)
B.b. W56	Vagina	2.36	57.8	2207	49	2	126027 / 46295	*	D (24 / 13)
B.b. N6D12	Vagina	2.29	58.5	2097	50	1	119740 / 29243	*	D (30 / 12)
B.b. 12–4	Vagina	2.26	58.7	2148	48	3	148702 / 48178	*	D (25 / 7)
B.b. ACS-071-V-Sch8b	Vagina	2.33	58.7	2046	53	4	NA	CP002743	C
B.b. JCM 1192^T^	Infant feces	2.27	58.9	2039	53	0	NA	AP012324	C
B.b. UCC2003	Infant feces	2.42	58.7	2131	54	3	NA	CP000303	C
B.b. JCM 7017	Infant feces	2.29	58.7	1995	54	2	NA	CP006712	C
B.b. JCM 7019	Adult feces	2.36	58.6	2133	56	2	NA	CP006713	C
B.b. NCFB 2258	Infant feces	2.32	58.7	2036	53	2	NA	CP006714	C
B.b. 689b	Infant feces	2.33	58.7	2052	53	0	NA	CP006715	C
B.b. S27	Infant feces	2.29	58.7	2005	53	2	NA	CP006716	C
B.b. CBT BR3	Infant feces	2.43	59.1	2195	54	2	NA	CP010413	C
B.b. LMC520	Infant feces	2.40	59.0	2146	55	1	NA	CP019596	C
***B*. *longum* (n = 26)**									
B.l.^#^ 239–2	Vagina	2.28	59.1	2060	50	8	33459 / 10497	*	D (60 / 56)
B.l. W35-1	Vagina	2.30	60.3	2133	49	1	17831 / 5459	*	D (81 / 132)
B.l. N2E12	Vagina	2.34	59.1	2114	51	5	80659 / 26287	*	D (43 / 12)
B.l. N2F05	Vagina	2.33	59.6	2076	55	2	105634 / 41749	*	D (30 / 13)
B.l. N2G10	Vagina	2.28	59.6	2200	38	0	9284 / 3230	*	D (165 / 186)
B.l. N3A01	Vagina	2.31	59.7	2056	51	5	82928 / 40450	*	D (33 / 13)
B.l. N3E01-2	Vagina	2.34	59.6	2167	51	3	202175 / 50971	*	D (23 / 1)
B.l. N5E04	Vagina	2.46	58.3	2219	45	0	154725 / 47782	*	D (27 / 3)
B.l. N6D05	Vagina	2.72	60.4	2641	77	3	25630 / 6992	*	D (80 / 105)
B.l.l. JCM 1217^T^	Infant feces	2.39	60.3	2090	73	1	NA	AP010888	C
B.l.l. JDM301	Gut	2.48	59.8	2156	55	1	NA	CP002010	C
B.l.l. BBMN68	Elderly feces	2.27	59.9	1959	54	3	NA	CP002286	C
B.l.l. KACC 91563	Infant feces	2.40	59.8	2064	56	1	NA	CP002794	C
B.l.l. GT15	Adult feces	2.34	60.0	2021	56	1	NA	CP006741	C
B.l.l. NCIMB 8809	Human feces	2.34	60.1	2037	56	1	NA	CP011964	C
B.l.l. CCUG 30698	Gut	2.46	60.2	2184	72	1	NA	CP011965	C
B.l.l. AH1206	Infant feces	2.42	60.2	2179	60	2	NA	CP016019	C
B.l. NCC2705	Infant feces	2.26	60.1	1799	57	1	NA	AE014295	C
B.l. DJO10A	Adult feces	2.39	60.1	2105	58	2	NA	CP000605	C
B.l. 105-A	Human feces	2.29	60.1	1950	56	2	NA	AP014658	C
B.l. BXY01	Gut	2.48	59.8	2158	55	1	NA	CP008885	C
B.l. BG7	Infant feces	2.46	60.0	2128	57	1	NA	CP010453	C
B.l. 35624	Gut	2.26	60.0	1942	57	2	NA	CP013673	C
B.l.i. JCM 1222^T^	Infant feces	2.83	59.9	2673	77	0	NA	CP001095	C
B.l.i. 157F	Infant feces	2.41	60.1	2147	59	1	NA	AP010890	C
B.l.i. BT1	Infant feces	2.58	59.4	2308	56	1	NA	CP010411	C

^#^ B.b. = *B*. *breve*; B.l. = *B*. *longum*; B.l.l. = *B*. *longum* subsp. *longum*; B.l.i. = *B*. *longum* subsp. *infantis*.

* Genome sequenced as part of this study. ^T^ = type strain. NA = not applicable. C = complete, D = draft.

### Genome sequencing and assembly

Libraries were prepared with 1 ng of genomic DNA using the Nextera XT DNA Library Preparation Kit (Illumina Inc., San Diego, CA) according to the manufacturer’s instructions. After PCR amplification and clean up, the fragment size distribution of the tagmented DNA was analyzed using the High Sensitivity DNA Analysis Kit on Agilent 2100 Bioanalyzer (Agilent Technologies, Santa Clara, CA). PhiX DNA (15% (v/v)) was added to the pooled indexed libraries prior to loading onto the flow cell. The libraries were sequenced using Reagent Kit V2 (500 cycles) on Illumina Miseq platform (Illumina Inc., San Diego, CA).

Raw sequence reads were trimmed for quality using Trimmomatic [37] with a minimum read length of 40 and quality cut-off of Phred score of 20. To estimate genome coverage and calculate average insert size, reads were mapped on to the reference genome of *B*. *breve* (Genbank Accession AP012324) or *B*. *longum* (Genbank Accession NC_015067.1) using Bowtie2 [38] and the results were converted to BAM format for viewing in Qualimap v2.2.1 [39]. High quality reads were assembled with SOAPdenovo2 [40] using the estimated average insert size from Qualimap analysis.

### Genome analysis

Genomes sequenced in this study were annotated using the NCBI (National Center for Biotechnology Information) Prokaryotic Genome Automatic Annotation Pipeline (PGAAP). For all other genomes, the published annotation from the NCBI Genbank or Refseq database was used. CRISPRFinder (http://crispr.i2bc.paris-saclay.fr/) was used to identify CRISPR (clustered regularly interspaced short palindromic repeats) within the genome sequences [41]. Annotated genomes were also submitted to the Joint Genome Institute (JGI - http://jgi.doe.gov/) for COG (Clusters of Orthologous Groups) category assignment.

Overall genome similarities were assessed by calculating the Average Nucleotide Identity by Mummer (ANIm) and tetranucleotide scores (tetra) within JSpecies [42]. ANIm values were visualized as heatmap, generated in R. The ‘*vegdist*’ function was used to calculate the Euclidean distance between the ANI divergence values, and ‘*hclust*’ function was used to calculate the complete linkage on the distance matrix, in R.

CSI Phylogeny was used to call SNPs (single-nucleotide polymorphisms) and infer phylogeny based on the concatenated alignment of SNPs. The following settings were used: minimum depth at SNP positions of 10; relative depth at SNP positions of 10; minimum distance between SNPs (prune) of 10; minimum SNP quality of 30; minimum read mapping quality of 25; minimum Z-score: 1.96. A maximum likelihood tree indicating the whole genome phylogeny was also computed within CSI Phylogeny [43].

Pangenome calculation based on a pairwise BLASTp comparison of all predicted proteins from all genomes and rarefaction plots were conducted using the R package micropan [44]. Gene clusters (families) were identified using complete linkage and the resulting table of gene cluster prevalence in individual genomes was used to determine the size of the core genome for each species and the distribution of gene clusters among genomes from gut and vaginal isolates. Gene cluster prevalence data was converted to a binary matrix and similarities in presence/absence patterns were calculated using the Jaccard index. Dendrograms were calculated using the unweighted pair group method with arithmetic mean (UPGMA) in DendroUPGMA ([45] http://genomes.urv.cat/UPGMA/index.php).

## Results and discussion

### General genome features

We performed a comparative genomic analysis between gut and vaginal isolates for two *Bifidobacterium* species commonly found in the human gut and vagina: *B*. *breve* and *B*. *longum*. Thus, all analysis comparing vaginal and gut strains was performed in parallel for these two species. The general features of all genomes included in this study are listed in Table 1.

Seventeen *B*. *breve* genomes were analyzed, seven of which were sequenced in this study. We also analyzed the genome sequences of twenty-six *B*. *longum* strains, nine of which were sequenced as part of this study. Genome sequence data and annotated assemblies have been deposited in GenBank under BioProject PRJNA387952. The *B*. *breve* genomes were sequenced to an average of 97-fold coverage ± 47 (range 14–270), and *B*. *longum* genomes were sequenced to an average of 57-fold coverage ± 34 (range 11–182). Currently, *B*. *longum* encompass three subspecies: -longum, -infantis and -suis, but it has been previously considered as three separate species (*B*. *longum*, *B*. *infantis* and *B*. *suis*) or as a unified species (*B*. *infantis* and *B*. *suis* were published as synonyms of *B*. *longum*) [46, 47]. Considering this controversial taxonomic history of *B*. *longum*, we opted to include in our analysis published complete genomes of *B*. *longum* of gut origin regardless of subspecies, which included (n = 8 subsp. *longum*, n = 3 subsp. *infantis* and n = 6 for which no subspecies affiliation was reported).

**Fig 1:**
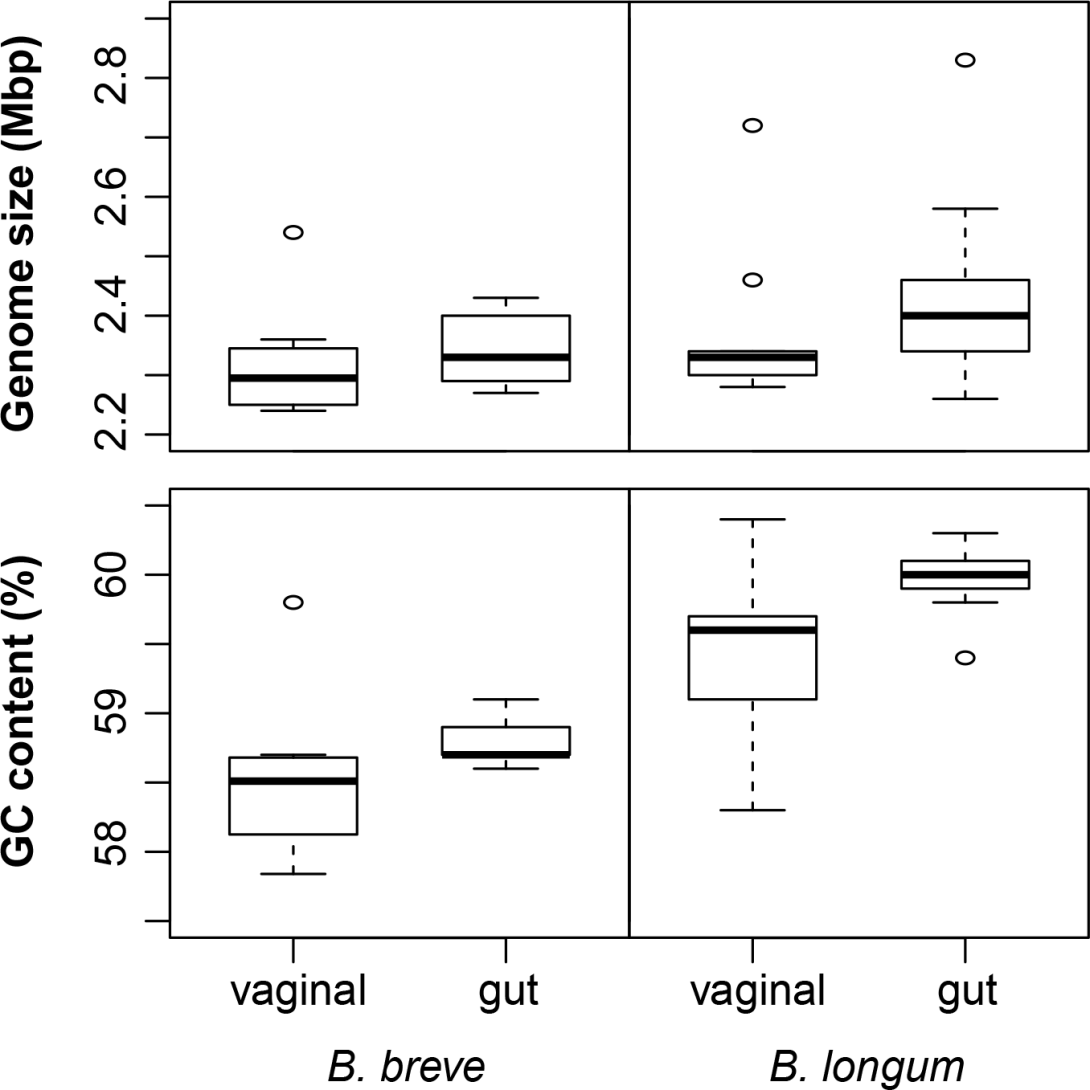
Genome features. Genome size and GC content of *B*. *breve* and *B*. *longum* of gut and vaginal origin.

The average genome size of vaginal and gut *B*. *breve* was 2.32 ± 0.09 Mb and 2.34 ± 0.05 Mb, respectively; where vaginal strains 91–1 and 322–1 were the smallest (2.24 Mb) and the vaginal strain 30–1 was the largest (2.54 Mb). For *B*. *longum*, the average genome sizes of vaginal and gut isolates were 2.37 ± 0.14 Mb and 2.41 ± 0.13 Mb, respectively; the smallest genomes were represented by gut isolates NCC2705 and 35624 (2.26 Mb) and the largest genome was from JCM 1222^T^ (2.83 Mb). All genomes analyzed had high GC content, a known characteristic of the genus *Bifidobacterium* and previously reported as 55–67 mol% [5]. Vaginal and gut *B*. *breve* have 58.5% and 58.8% genomic GC content, respectively; and vaginal and gut *B*. *longum* have 59.5% and 60% of GC content, respectively. There were no differences in genome size and GC content between gut and vaginal isolates, either for *B*. *breve* or *B*. *longum* (t-test, all p>0.05) (Fig 1).

The genomes of *B*. *breve* and *B*. *longum* contained an average of 2113 and 2137 genes, respectively, which is within the range of number of predicted genes previously reported for *Bifidobacterium* spp. (1369–2564 genes) [48]. Also, *B*. *breve* and *B*. *longum* contained an average of 1.6 (range 0–4) and 1.8 (range 0–8) CRISPR, respectively. The inclusion of complete and draft genomes in the analysis and the choice of particular analysis tool may have affected the CRISPR analysis; however, there was no difference in the number of genes and CRISPR between gut and vaginal isolates of either *B*. *breve* or *B*. *longum* (t-test, all p>0.05). Since gut strains are more likely to be often exposed to viral infection, a greater CRISPR activity might be expected within the gut isolates, which was not the case. CRISPR have been implicated in chromosomal rearrangement, modulation of expression of neighbouring genes, target for DNA binding proteins, and DNA repair [49]. More recently, it has been shown to act as the defense mechanism in bacteria against phages and plasmids by providing adaptive immunity [50]. Notably, vaginal *B*. *longum* 239–2 and N2E12 had a total of 8 and 5 CRISPR, respectively, which suggest these strains had an active CRISPR immune system against potentially damaging foreign DNA. Previous studies have shown that CRISPR systems are frequent and diverse in the genus *Bifidobacterium* and differences in the frequency of CRISPR-Cas systems (CRISPR and CRISPR-associated proteins) within species are an indication that CRISPR distribution is strain-dependent [51, 52].

**Fig 2:**
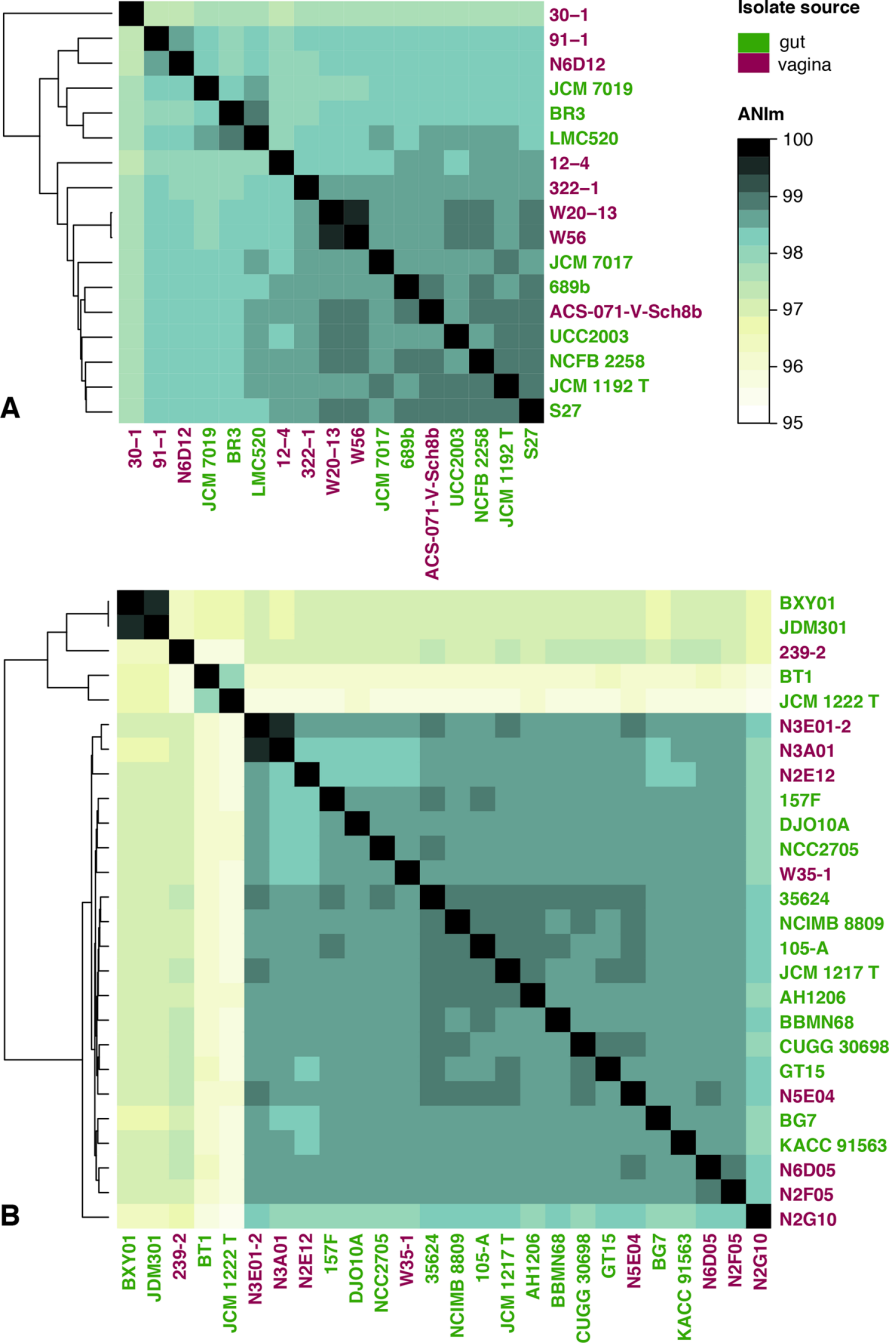
Average nucleotide identity (ANI). Heatmap of ANI values between genomes of gut and vaginal origin. **(A)** *B*. *breve*; **(B)** *B*. *longum*. T = type strain.

The overall genome sequence similarity between vaginal and gut strains was assessed based on average nucleotide identity (ANI). A hierarchal clustering based on the distance matrix of ANI divergence values was computed and visualized as a heatmap (Fig 2). All ANI values between isolates of the same species were >95%, consistent with their identification as members of the same species [53]. The genomes of vaginal *B*. *breve* did not cluster separately from the gut isolates (Fig 2A). Two clusters of *B*. *longum* were apparent but these do not correspond to subspecies *longum* or *infantis* based on genomes where a subspecies was identified (Fig 2B). For example, in the upper cluster of five genomes, JDM301 is reported to be subsp. *longum*, JCM 1222^T^ and BT1 are reported to be subsp. *infantis* and no subspecies information is available for 239–2 or BXY01 (Table 1). This result is not unexpected since genome-wide ANI may not provide sufficient resolution to discern subspecies and identities between the two *B*. *longum* clusters were >95%. Similarly, vaginal *B*. *longum* did not form a separate cluster from the gut isolates (Fig 2B). Therefore, vaginal and gut isolates could not be distinguished based on their overall nucleotide identity, either for *B*. *breve* or *B*. *longum*. The relatedness of isolates was also assessed by calculating the tetranucleotide scores (tetra), which led to similar results as ANI (data not shown).

### Whole genome phylogeny

**Fig 3:**
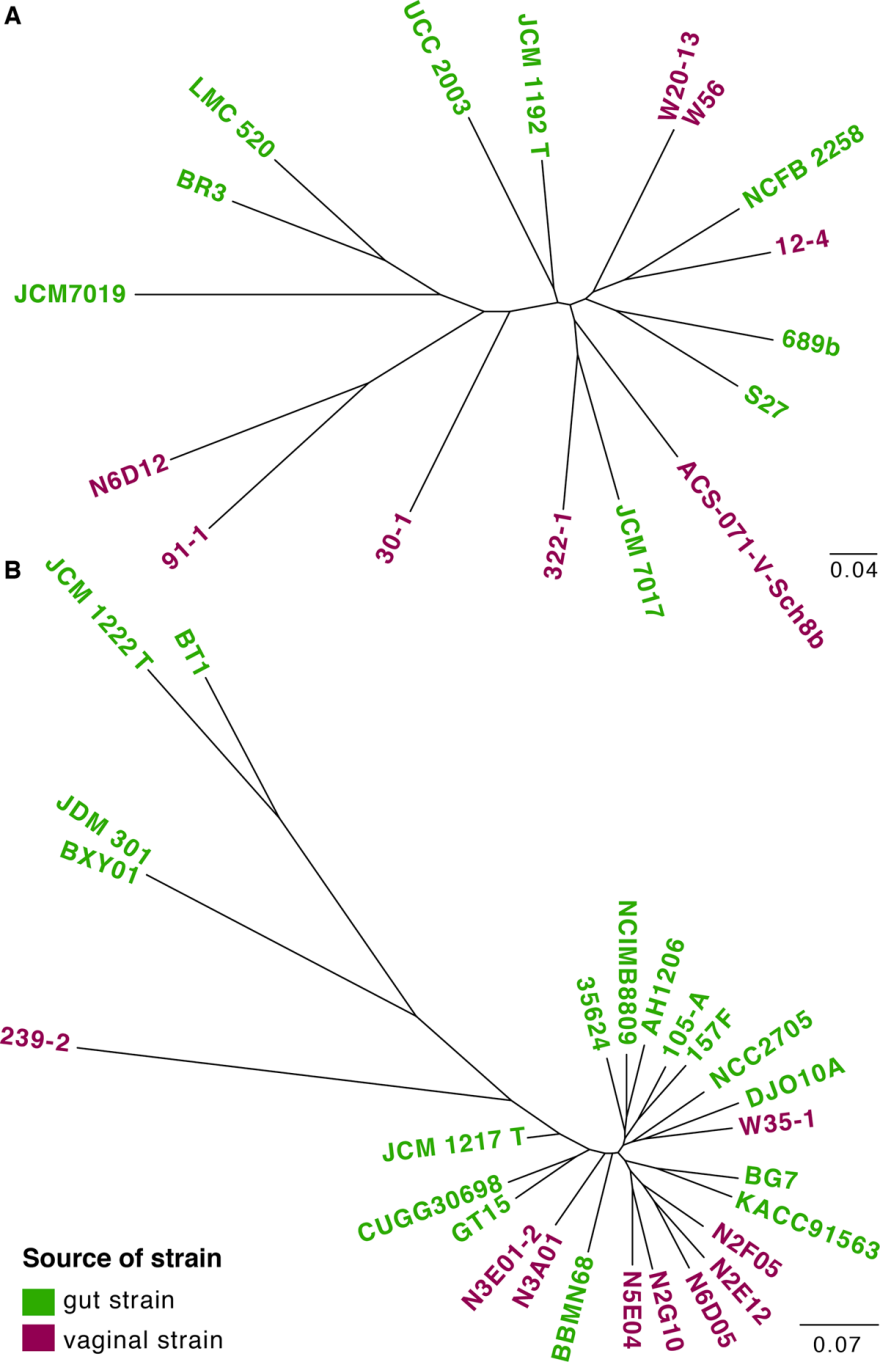
Whole genome phylogenies based on SNPs. **(A)** *B*. *breve;* **(B)** *B*. *longum*. T = type strain.

Whole genome phylogeny of isolates was inferred based on the concatenated alignment of SNPs, and a maximum likelihood circular tree was created for phylogeny visualization (Fig 3). The comparison of all genomes revealed an average of 9307 SNPs in *B*. *breve* strains and an average of 9050 SNPs in *B*. *longum* strains. Phylogenetic analysis indicated that vaginal *B*. *breve* isolates did not cluster separately from the gut isolates (Fig 3A). Similarly, the genomes of vaginal *B*. *longum* did not form a separate cluster from the genomes of gut strains (Fig 3B).

### COG distribution

**Fig 4:**
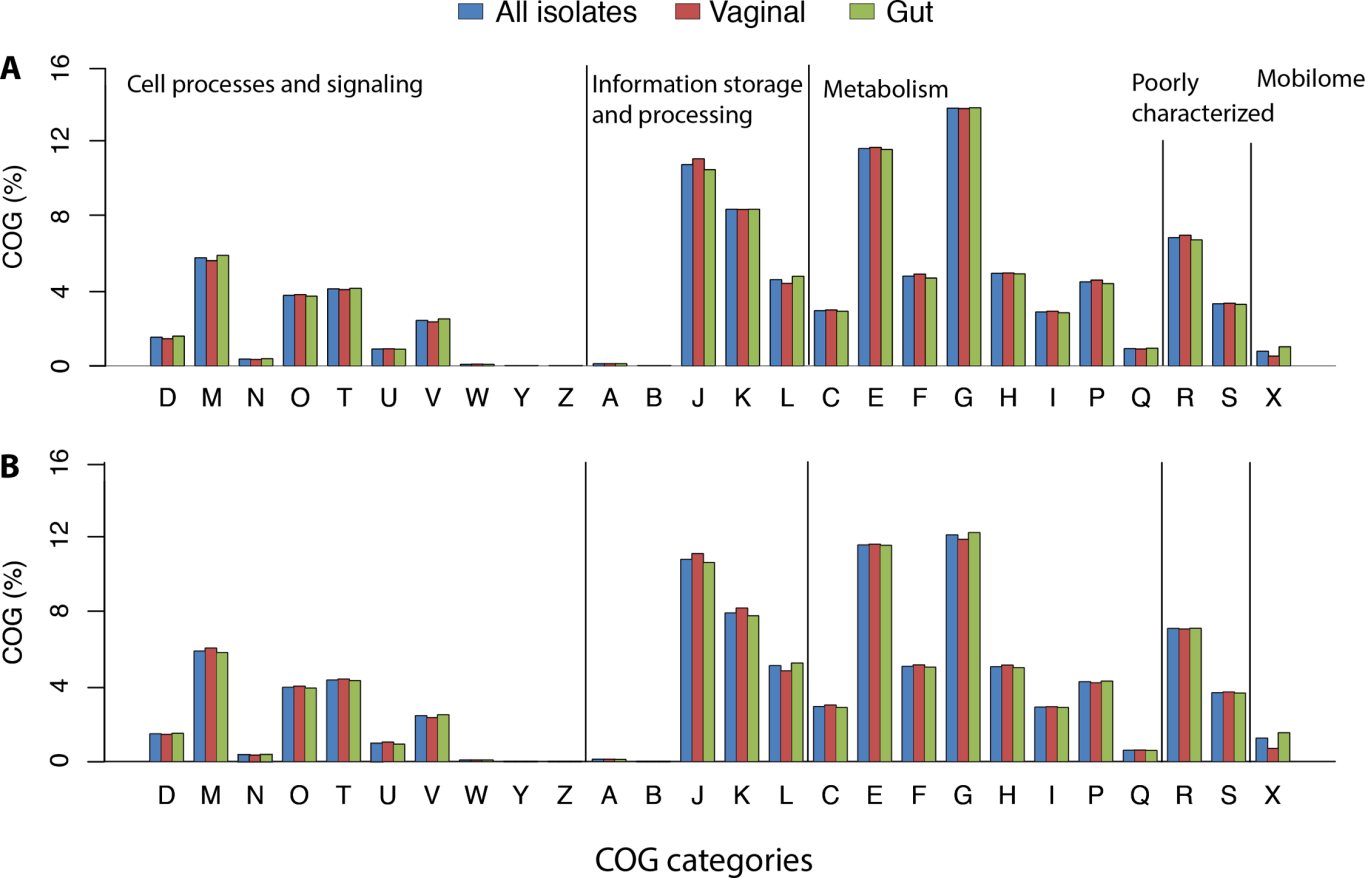
Clusters of Orthologous Groups (COG). COG function distribution among genomes of **(A)** *B*. *breve* and **(B)** *B*. *longum*. COG classification: [D] Cell cycle control, cell division, chromosome partitioning; [M] Cell wall/membrane/envelope biogenesis; [N] Cell motility; [O] Post-translational modification, protein turnover, and chaperones; [T] Signal transduction mechanisms; [U] Intracellular trafficking, secretion, and vesicular transport; [V] Defense mechanisms; [W] Extracellular structures; [Y] Nuclear structure; [Z] Cytoskeleton; [A] RNA processing and modification; [B] Chromatin structure and dynamics; [J] Translation, ribosomal structure and biogenesis; [K] Transcription; [L] Replication, recombination and repair; [C] Energy production and conversion; [E] Amino acid transport and metabolism; [F] Nucleotide transport and metabolism; [G] Carbohydrate transport and metabolism; [H] Coenzyme transport and metabolism; [I] Lipid transport and metabolism; [P] Inorganic ion transport and metabolism; [Q] Secondary metabolites biosynthesis, transport, and catabolism; [R] General function prediction only; [S] Function unknown; [X] Mobilome: prophages, transposons.

To identify significant differences in the predicted functional repertoires of gut or vaginal isolates of *B*. *breve* or *B*. *longum*, predicted proteins were functionally categorized based on COG (Cluster of Orthologous Group) assignment and the proportions in each category were compared between vaginal and gut isolates (Fig 4). Most sequences in *B*. *breve* and *B*. *longum* were assigned to the ‘carbohydrate transport and metabolism’ category, followed by ‘amino acid transport and metabolism’ and ‘translation, ribosomal structure and biogenesis’ categories, which is consistent with previous reports [54, 55]. Most importantly, vaginal and gut strains did not differ in terms of COG category distribution. This does not mean that there are not differences in the specific genes within these larger functional categories, but only that the proportion of each genome encoding functions in the broad categories are similar. Considering that carbohydrates are less abundant in quantity and variety in the vagina relative to the gut, we had anticipated that if vaginal isolates have genome adaptations, these would include presence of fewer genes involved in metabolism. Our observation of similar proportions of COGs in category G (‘carbohydrate transport and metabolism’) in vaginal and gut isolates corroborates our previous observation that the carbohydrate utilization profile phenotypes of the vaginal isolates included in this study do not differ from those reported for the type strains of *B*. *breve* and *B*. *longum* [19].

COGs were also investigated in terms of their presence/absence among isolates. *B*. *breve* isolates were represented by a set of 1016 COG, 489 of which were present in all strains (vaginal and gut origin). A total of 30 COG were exclusively present in vaginal isolates, i.e., they were absent in gut isolates and present in at least one vaginal isolate. Notably, the prevalence of these 30 “unique” COG among vaginal isolates was low, ranging from 12.5% (1/8) to 37.5% (3/8), suggesting it is unlikely that these rare COG represent a biologically significant vaginal adaptation. Similarly, a total of 34 COG were exclusively present in gut isolates, but their prevalence was also mostly low, ranging from 11.1% (1/9) to 33.3% (3/9), with only one exception: COG1396 (Transcriptional regulator, contains XRE-family HTH domain) that was present in 55.5% (5/9) of the gut isolates. Two additional COG showed substantial differences in distribution between vaginal and gut *B*. *breve* isolates. Glucan phosphorylase (COG0058) was present in only 12.5% (1/8) of vaginal isolates against 88.9% (8/9) of isolates of gut origin. On the other hand, the predicted ABC-type sugar transport system (permease component) (COG4158) was present in 87% (7/8) and 22.2% (2/9) of vaginal and gut isolates, respectively.

For *B*. *longum*, a total of 1128 COG were identified, 451 of which were present in all strains (vaginal and gut). Although 26 COG were exclusively present in vaginal isolates, most of them were found in only one or two vaginal isolates, with one exception: COG3695 (Alkylated DNA nucleotide flippase Atl1), which was present in 55.5% (5/9) of vaginal isolates. On the other hand, a total of 113 COG were exclusively associated with isolates of gut origin, although their prevalence was also low, ranging from 5.8% (1/17) to 29.4% (5/17). The only exception was COG1672 (Predicted ATPase), which was found in 76.5% (13/17) of gut strains. Additionally, 5 COG were more frequently found in gut isolates than vaginal isolates: COG0481 (Translation elongation factor EF-4, membrane-bound GTPase), COG0802 (tRNA A37 threonylcarbamoyladenosine biosynthesis protein TsaE), COG1225 (Peroxiredoxin), COG0159 (Tryptophan synthase alpha chain), and COG0732 (Restriction endonuclease S subunit). Four COG were more prevalent in vaginal isolates: COG1327 (Transcriptional regulator NrdR, contains Zn-riB.b.on and ATP-cone domains), COG0328 (Ribonuclease HI), COG1983 (Phage shock protein PspC (stress-responsive transcriptional regulator)), and COG0759 (Membrane-anchored protein YidD, putatitve component of membrane protein insertase Oxa1/YidC/SpoIIIJ). Although there were differences in the presence/absence of COG between vaginal and gut isolates, differences were not systematically concentrated within function categories, and were insufficient to distinguish isolates from the two body sites.

### Pangenome analysis

**Fig 5:**
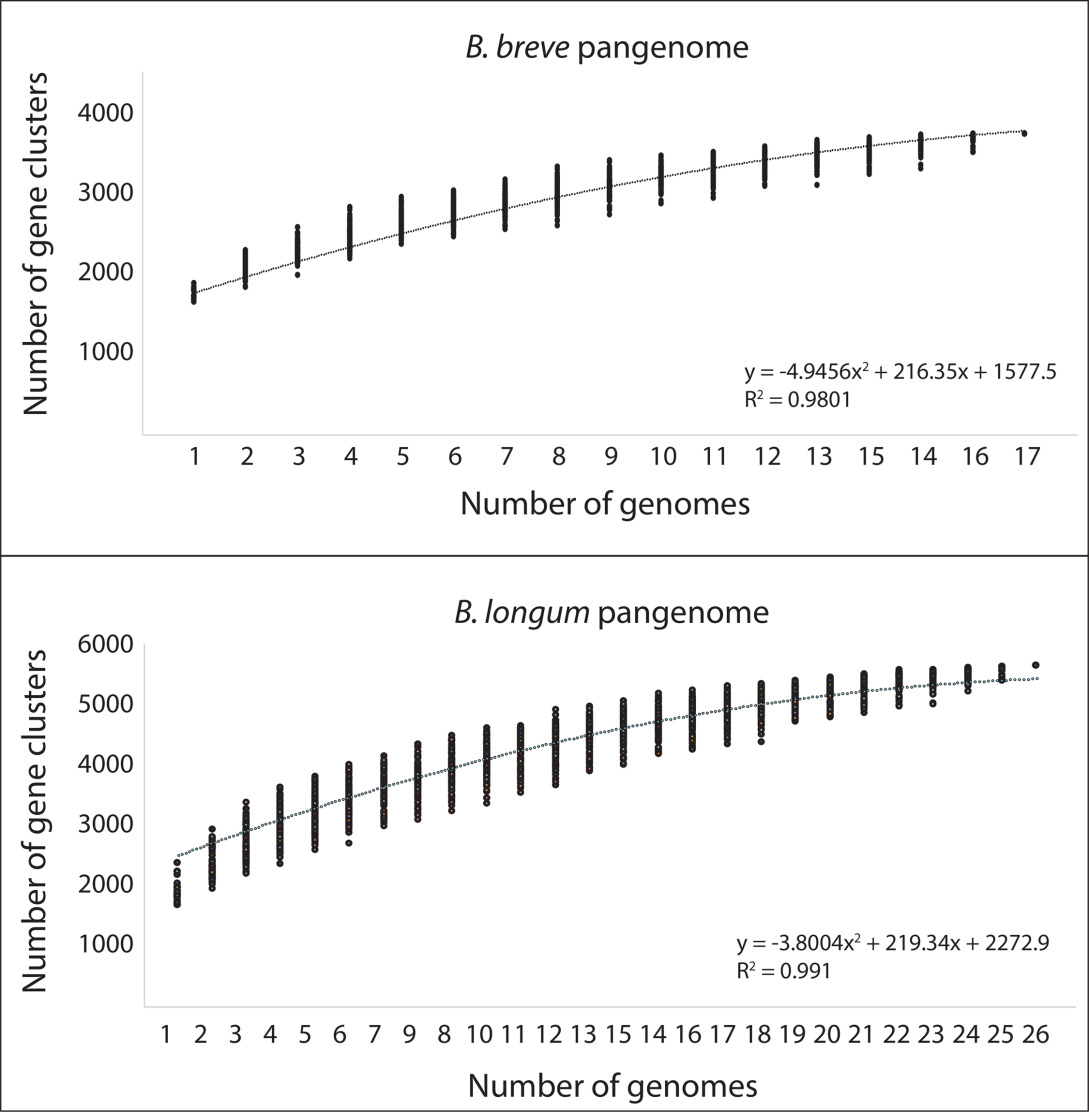
Rarefaction plot of *B*. *breve* and *B*. *longum* pangenome. Accumulated number of new gene clusters plotted against the number of genomes sequentially added. One hundred permutations were performed at each step.

The pangenome is defined as the entire gene set of all isolates, including genes present in all isolates (core genome) and genes present in one or some isolates (accessory genome). *B*. *breve* had a pangenome of 3773 gene clusters, consistent with a previous report (3667 in 13 genomes) [54] (Fig 5). The larger pangenome of *B*. *longum* (5609 gene clusters) reflects the inclusion of different *B*. *longum* subspecies in the analysis and is also consistent with a recent analysis of 20 *B*. *longum* genomes (5970 in 37 genomes) [56] (Fig 5). The content of pangenomes of *B*. *breve* and *B*. *longum* have recently been described in great detail [54–57] and so the focus of this analysis was on our primary objective, to determine if vaginal and gut bifidobacteria could be distinguished based on the presence/absence of components of the pangenome.

**Fig 6:**
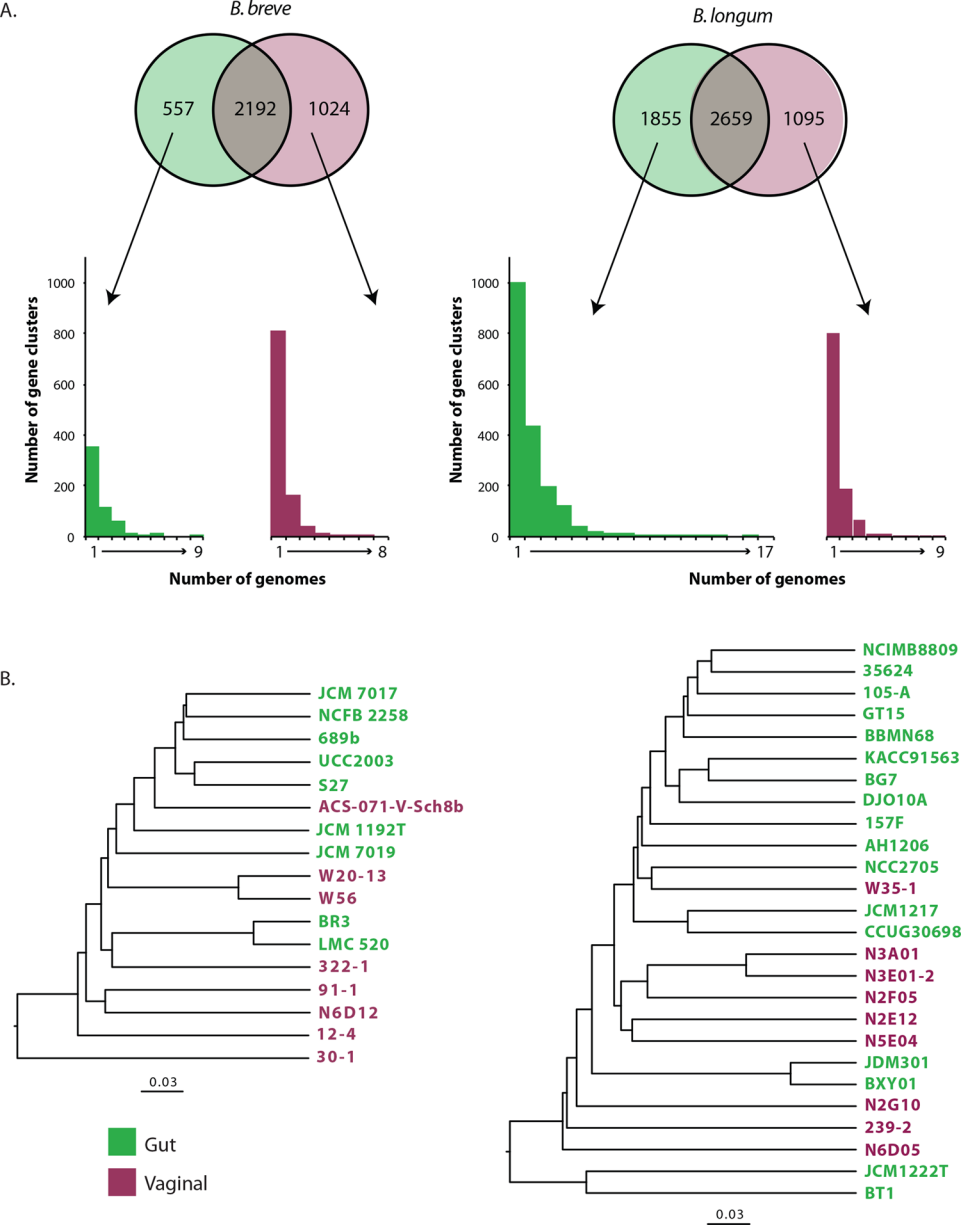
Shared and unique gene clusters. **(A)** The numbers of shared (present in at least one genome of each group) and unique (present in at least one genome of one group) gene clusters of *B*. *breve* and *B*. *longum* isolates from vaginal and gut microbiotas. Histograms below the Venn diagrams show the distribution of group-specific gene clusters among genomes and indicate that most group-specific gene clusters are found in only one genome. **(B)** Relationships of genomes based on presence/absence of gene clusters identified in the pangenomes for *B*. *breve* (3773 gene clusters) and *B*. *longum* (5609 gene clusters).

By strictest definition (gene must be present in all genomes included), the core genomes of *B*. *breve* and *B*. *longum* contained 916 and 835 gene clusters, respectively. There were 2192 gene clusters shared by at least one vaginal and one gut isolate of *B*. *breve*, while vaginal and gut *B*. *longum* had a total of 2659 shared gene clusters (Fig 6A). Relatively large numbers of gene clusters were identified exclusively in vaginal or gut bifidobacteria but further investigation showed that the majority of these were present in only one genome and thus could not be used to distinguish vaginal and gut isolates (Fig 6A). In fact, only two gene clusters in the *B*. *breve* pangenome, corresponding to a hypothetical protein and a putative fluoride ion transporter (crcB, Fluc family), were found in all (9/9) gut isolate genomes and none (0/8) of the vaginal isolate genomes. A crcB sequence was also found in 16/17 gut *B*. *longum* and 0/9 vaginal *B*. *longum* isolates. These small integral membrane proteins are important for counteracting toxicity of environmental fluoride anions and are widespread in bacteria [58]. Most of the gene clusters that were present in at least half of the genomes in one group and absent from genomes in the other group were unidentified (hypothetical proteins of unknown function), which is not surprising given that at least 20–40% of genes in sequenced bacterial genomes encode unknown functions [59]. Average numbers of gene clusters that were unique to one genome were 68.1 (range 6–247) for *B*. *longum* and 67.8 (range 2–228) for *B*. *breve*.

To determine if the vaginal and gut bifidobacteria genomes could be differentiated based on shared and unique gene clusters, hierarchical clustering of the presence/absence patterns of gene clusters in the calculated pangenome was conducted (Fig 6B). Similar to the results of the clustering based on ANI comparisons, the vaginal and gut bifidobacteria did not form separate clusters based on core and accessory genome content, which further emphasizes that although there is strain diversity in genome content, systematic differences that differentiate isolates based on source (vaginal or gut) are not apparent.

## Conclusions

In this study, we investigated the genomes of *B*. *breve* and *B*. *longum* from two different body sites of significant importance in neonatal health: gut and vagina. In all analyses, gut and vaginal strains of *B*. *breve* or *B*. *longum* were not distinguishable from each other based their genomic content. Consistent with these observations, it has been previously demonstrated that several vaginal and gut *Bifidobacterium* spp. did not differ based on phenotypic characteristics related to their carbohydrate fermentation patterns, lactic acid production and tolerance to low pH [19]. Our results support the hypothesis that vaginal and gut isolates represent the same bacterial population with similar genetic repertoires that allow them to efficiently colonize both body sites. The genomes included in our study are from isolates recovered from individuals of different ages around the world over many years. While this provides an opportunity to look at these species very broadly, an obvious complimentary study would be a comparison of gut and vaginal isolates from individual women, or paired samples from women and their babies. Both vaginal and gut microbiota are thought to contribute to mother-infant transmission of bifidobacteria, and recognition that vaginal and gut isolates represent the same population is an important step for future studies in maternal-neonatal health.
